# Investigating the role of Chinese sports media in shaping young adults’ exercise habits through the lens of the health belief model

**DOI:** 10.3389/fspor.2025.1600486

**Published:** 2025-09-22

**Authors:** Fangni Li

**Affiliations:** School of International Studies, Communication University of China, Beijing, China

**Keywords:** Chinese sports media, health belief model, exercise behaviour, digital fitness campaigns, health promotion

## Abstract

**Background:**

The rapid proliferation of Chinese sports media has significantly impacted young adults’ exercise habits, influencing their perceived susceptibility to health risks, perceived benefits of exercise, self-efficacy, and perceived barriers, according to the Health Belief Model (HBM). Hence, the research is based on the question: How are key components of the HBM (susceptibility, benefits, barriers, and self-efficacy) associated with exercise motivation among young adults exposed to Chinese sports media?

**Method:**

Utilizing a quantitative cross-sectional design and Structural Equation Modeling (SEM), this research analyzed data from 1,221 respondents to assess the psychological mechanisms underlying media-driven health behaviors.

**Results:**

Findings indicate that perceived susceptibility to health risks, perceived benefits of exercise, and self-efficacy are positively associated with higher levels of exercise motivation, while perceived barriers show a negative association. The findings demonstrated how media directly affects fitness views through its 4.81 average rating regarding the health dangers of inactivity. The study indicated that exercise increases physical health by an average score of 4.77 and high confidence levels in executing regular physical exercise activities. In confirming the study, the SEM evaluation tests yielded CFI = 1.000, SRMR = 0.022, and RMSEA = 0.002.

**Conclusion:**

Fitness influencers and government-led efforts in Chinese sports media are associated with higher reported motivation to exercise despite unrealistic body standards and accessibility inequities. This study helps policymakers, media strategists, and public health experts connect media influence and behavioral health to improve digital fitness initiatives’ media-driven health promotion. Future research should explore AI-driven personalized fitness interventions leveraging Chinese sports media analytics.

## 1 Introduction

The rapid expansion of digital media has transformed how societies engage with sports, influencing viewership and participation in physical activity (1, 2). Digital platforms now make sports-related content widely available, thus creating an exceptional opportunity to boost fitness awareness and promote physical activity. The global presence of sedentary lifestyles continues to be a significant health issue because it produces rising levels of obesity alongside heart diseases and mental health struggles (1, 3). Sports media, as a strong influence instrument, can form public perceptions regarding exercise and health behaviors within the Chinese domain. As a significant digital sports media consumer and producer, China is crucial in establishing fitness standards of practice nationwide (4, 5). The digital platforms of Weibo, Douyin (TikTok), and WeChat now act as the main channels through which the Chinese public discusses health and sports matters. The “Healthy China 2030” strategy receives governmental support through media campaigns that target young adult exercise behaviors. Scholarly investigation of Chinese sports media's effects on young people's exercise behaviors remains limited due to insufficient research. Despite widespread engagement with digital sports platforms, little is known about how psychological health beliefs interact with media messaging to influence exercise behavior in Chinese youth (6, 7). As a psychological model, the Health Belief Model (HBM) provides systematic structures for investigating the behavioral effects of media intake on exercise habits (8).

The global sports media landscape has changed from essential television broadcasting to digital networks that host machine-generated content, user-generated content, and interactive platforms. Sports-related content now spreads through ESPN and CCTV-5 in addition to Tencent Sports and social media influencers who serve as key platforms for delivery (9, 10). Current arguments persist over the effects of media influence on public health. Sports media provides inspirational opportunities for active living, yet it often maintains unattainable body expectations, which may cause exercise-related stress or dropouts from fitness activities. The sports media industry in China functions under a specific socio-political system whose government standards influence how content spreads throughout the nation (11, 12). Fitness campaigns launched by the general administration of sport work to promote exercise, yet misleading information about fitness commercialized trends and unachievable fitness objectives continue to create obstacles for Chinese citizens (11, 13). Furthermore, media access gaps between urban and rural regions determine the media sports can utilize to advertise exercise, necessitating the study of the broader media consumption effects across different populations (14, 15).

The rationale for this research is the increasing worry about physical inactivity among young adults in China and the world. This research responds to rising concerns about sedentary lifestyles among young Chinese adults despite the digital proliferation of health-related media. While the HBM has been applied extensively in Western contexts, few studies explore its relevance within China's socio-political environment and digital media ecosystem. This study adapts the HBM by examining how centrally controlled fitness messaging, influencer-led fitness culture, and varying access to sports media shape health perceptions and exercise behavior. Rather than offering a novel theoretical extension of HBM, this study contributes by contextualizing it within a specific cultural and media landscape, emphasizing its adaptability and practical relevance in non-Western societies.

This study applies the HBM to a media-driven, culturally specific setting by examining the mechanisms through which Chinese sports media influences health-related beliefs and exercise intentions. While prior research has used the HBM in public health campaigns and education, our study applies it to digital sports media platforms such as Weibo and Douyin, where health messaging is filtered through both state-controlled narratives and influencer-driven content. This study contributes in three specific ways; first, we extend the HBM's utility by situating it within a media ecology shaped by both governmental policy (e.g., Healthy China 2030) and market-driven influencer culture. Second, the study empirically tests how media content aligns with or distorts traditional HBM constructs in a Chinese context. Third, we provide actionable insights for media strategists and public health officials on how to tailor exercise-promoting content to enhance perceived benefits and self-efficacy while minimizing perceived barriers.

The aim is to understand how Chinese sports media influences exercise-related health beliefs among young adults, using HBM as an analytical lens. By focusing on perceived susceptibility, benefits, barriers, and self-efficacy, we assess how digital content shapes exercise intentions. Recognizing the limitations of a cross-sectional approach, we interpret these findings as correlational and call for further research using longitudinal or experimental designs to evaluate long-term behavioral impact. This study addresses two guiding questions: 1. how are HBM constructs (perceived susceptibility, benefits, barriers, and self-efficacy) associated with exercise motivation in the context of Chinese sports media? 2. How does the media's structure in China (marked by government messaging and influencer dynamics) shape these health beliefs? These questions are grounded in the theoretical relevance of the HBM and a practical need to optimize digital fitness communication strategies in China.

## 2 Methodology

### 2.1 Research design

The deductive study evaluated how media exposure affects exercise practices by testing predetermined hypotheses based on the HBM. A standardized questionnaire measured key characteristics such as self-efficacy, exercise habits, perceived barriers, perceived advantages, and perceived susceptibility (16). A 6-point Likert scale, 1 denoting “strongly disagree” and 6 denoting “strongly agree,” was used to operationalize these variables to capture respondents' sentiments without a neutral midpoint and guarantee more distinct results (17). This design captures a single-time-point association between digital media exposure and self-reported health beliefs and exercise intentions. Because the study uses a cross-sectional survey, no claims can be made about the direction or causality of observed relationships. While associations are reported, longitudinal or experimental designs would be required to determine whether media exposure influences exercise behavior over time. Adjusting for demographic characteristics, including age, gender, education level, and exercise frequency, made it easier to examine statistical correlations between independent and dependent variables (18). By following ethical research guidelines, the study ensured that participants gave informed consent and participated willingly. Confidentiality and anonymity were preserved; no personally identifying information was used to analyze or safely store the data. The participants' privacy was protected by following Chinese data protection laws using Wenjuanxing for data gathering (19). Importantly, this study's cross-sectional design does not permit causal interpretation. The observed associations between media exposure and HBM constructs reflect correlation at a single point in time.

### 2.2 Sampling and population

The target population in China is adults between 18 and 30 years old. This group was chosen because of its high use of digital sports media and propensity for sedentary lifestyles associated with work and school obligations. Participants were selected from various backgrounds to guarantee coverage across several behavioral and demographic categories, such as education level and frequency of exercise. A stratified random sampling procedure was used to improve the sample's representativeness (20). The survey link was shared on social media, academic networks, and online forums devoted to fitness using digital channels that the target audience commonly uses. The study successfully obtained 1,221 valid responses, above the bare minimum needed for statistical power in analyses based on SEM.

### 2.3 Data collection

Wenjuanxing (https://www.wjx.cn/), a well-known online survey platform in China that guarantees safe, extensive participant recruitment and real-time data gathering, was the only method to obtain the data. Respondents gave their informed consent before filling out the survey and willingly participated. IP address limitations and time constraints per questionnaire submission were among the steps to avoid duplicate responses (14, 15). There were four main sections to the questionnaire. Demographic information such as age, gender, education level, and frequency of exercise were recorded in the first section. The second segment examined how people consume sports media, including how often they are exposed, what media they consume, and how they interact with sports influencers and government-led fitness initiatives (18).

### 2.4 Data analysis

SEM was performed using R, enabling a sophisticated examination of the complex relationships among media exposure, health beliefs, and exercise behaviors. SEM was selected due to its capacity to simultaneously assess direct and indirect relationships between latent variables while accounting for measurement errors (21). Before conducting SEM, data were cleaned and preprocessed, including removing incomplete responses and assessing multivariate normality. Descriptive statistics were calculated to summarize respondents' demographics, media consumption patterns, and exercise habits. Internal consistency of the measurement scales was evaluated using Cronbach's Alpha, which indicated excellent reliability with a value of 0.94. The 95% confidence interval (CI) for Cronbach's Alpha ranged from 0.94 to 0.95, further confirming the robustness of the measurement scales (22, 23).

The study presents the reliability analysis of the scale items used to measure the key constructs in the study, including perceived susceptibility to health risks, perceived benefits of exercise, self-efficacy, perceived barriers to exercise, and media influence on exercise habits. The reliability of these constructs is assessed using Cronbach's Alpha, the 95% CI for Alpha (Feldt method), and the average inter-item correlation. The overall Cronbach's Alpha for the scale items is 0.94, indicating excellent internal consistency (24). A value above 0.9 suggests that the items within each construct are highly correlated, making the measurement reliable for assessing the intended psychological and behavioral constructs related to exercise habits. The CI for Cronbach's Alpha ranges from 0.94 to 0.95, reinforcing the robustness of the reliability estimate. The narrow range within this high reliability level further supports the consistency of the scale. The average inter-item correlation is 0.35, which falls within the recommended range (0.15–0.50) (25).

## 3 Results

The findings provided strong empirical support for the theoretical framework and research hypotheses. Table 1 displays descriptive data for the items reflecting perceived sensitivity to health hazards, including mean, standard deviation (SD), minimum and maximum values, skewness, and kurtosis. These numbers provide insight into how respondents see the detrimental health impacts of physical inactivity, particularly in relation to media exposure. The majority of respondents clearly agree with the assertions about the negative effects of inactivity on one's health, as seen by the mean scores for each question, which range from 4.40 to 4.81. The SDs, which vary from 1.31 to 1.51, indicate that while the majority of respondents believe they are susceptible to health problems, there are some differences in perceptions. The negative skewness of all the questions (varying from −1.06 to −0.95) indicates that responses are skewed in favor of higher levels of agreement. This implies that the majority of respondents are aware of the negative health effects of physical inactivity. From 0.00 to 0.43, the kurtosis values show that the response distribution is about normal. This suggests that although responses are slightly biased towards agreement, there are neither extreme outliers nor heavy-tailed distributions.

**Table 1:** Descriptive statistics for perceived susceptibility to health risks.

Perceived susceptibility to health risks	Mean	SD	Min	Max	Skew	Kurtosis
I believe a lack of regular exercise significantly increases the likelihood of developing health problems.	4.81	1.44	1	6	−1.06	0.16
Chinese sports media content makes me more aware of the health risks associated with a sedentary lifestyle.	4.4	1.4	1	6	−0.96	0.21
I feel personally at risk of health issues due to my current level of physical inactivity.	4.72	1.51	1	6	−1.02	0
Media reports on health concerns prompt me to reflect on my exercise habits.	4.42	1.35	1	6	−0.95	0.27
I frequently consider the long-term health impacts of not exercising regularly.	4.43	1.36	1	6	−1	0.37
Stories about health risks in Chinese sports media align with my personal concerns.	4.46	1.31	1	6	−0.98	0.43

Table 2 presents descriptive statistics for the items measuring Perceived Benefits of Exercise, including mean, SD, minimum and maximum values, skewness, and kurtosis. These metrics provide insight into respondents' beliefs about the advantages of exercise, particularly as influenced by Chinese sports media. The majority of respondents agree with the statements on the benefits of exercise, according to the mean values, which range from 4.36 to 4.77. The SDs, which show some variance in sample perception and range from 1.33 to 1.48, indicate moderate response variability. All items have negative skewness (ranging from −1.06 to −0.91), suggesting that responses are biassed towards higher levels of agreement. Sports media reports that most responders seem to concur that exercise has a number of benefits. Kurtosis scores vary from 0.14 to 0.32, indicating that the distributions are largely normal and free of high peaks or outliers. This implies that the variation is still balanced even though the replies are somewhat centred around agreement.

**Table 2:** Descriptive statistics for perceived benefits of exercise.

Perceived benefits of exercise	Mean	SD	Min	Max	Skew	Kurtosis
Regular exercise, as highlighted in Chinese sports media, improves my physical health.	4.77	1.48	1	6	−1.06	0.14
Chinese sports media effectively demonstrates the mental health benefits of regular exercise.	4.44	1.36	1	6	−0.96	0.27
Media portrayals of exercise encourage me to believe it improves my appearance.	4.39	1.39	1	6	−0.94	0.16
I associate exercise with social benefits, such as better relationships, as shown in sports media stories.	4.39	1.37	1	6	−0.97	0.29
Success stories of individuals in Chinese sports media motivate me to exercise.	4.36	1.34	1	6	−0.91	0.17
Watching fitness-related media reinforces my belief in the positive outcomes of regular exercise.	4.41	1.33	1	6	−0.97	0.32

Table 3 presents the descriptive statistics for Self-Efficacy, which measures respondents' confidence in their ability to engage in regular exercise. The mean, SD, minimum and maximum values, skewness, and kurtosis provide insights into how self-efficacy is influenced by personal determination and media exposure. The mean scores, which range from 4.39 to 4.46, indicate that most respondents agree with statements about their confidence in frequently exercising. There is a significant amount of variety in the replies, with some respondents feeling more confident than others, according to the SDs, which vary from 1.33 to 1.40. All of the questions have negative skewness, ranging from −1.03 to −0.89. This indicates that most replies are grouped in the direction of agreement and that fewer people disagree. With the most negative skew (−1.03), this statement, “Chinese sports media inspires me to believe I can overcome barriers to exercising,” shows that a sizable percentage of respondents strongly agree with it. The kurtosis values, which vary from 0.10 to 0.44, show that the responses are somewhat scattered about the mean. The statement with the greatest kurtosis (0.44) is “Chinese sports media inspires me to believe I can overcome barriers to exercising,” indicating that the majority of responses revolve around agreement.

**Table 3:** Descriptive statistics for self-efficacy.

Self-efficacy	Mean	SD	Min	Max	Skew	Kurtosis
I am confident that I can regularly engage in exercise, even with a busy schedule.	4.39	1.4	1	6	−0.92	0.1
Chinese sports media inspires me to believe I can overcome barriers to exercising.	4.45	1.33	1	6	−1.03	0.44
I believe I can maintain an exercise routine, even with limited access to fitness resources.	4.44	1.35	1	6	−0.98	0.34
I feel capable of following exercise routines promoted in sports media content.	4.46	1.33	1	6	−0.94	0.32
Watching exercise demonstrations in Chinese sports media increases my confidence to try similar activities.	4.46	1.34	1	6	−0.98	0.31
Chinese sports media makes exercise seem achievable for someone like me.	4.39	1.34	1	6	−0.89	0.21

Table 4 displays the descriptive data for Perceived Barriers to Exercise, a measure of how much people believe they cannot exercise consistently despite the influence of the media. The table shows the SD, mean, skewness, kurtosis, and lowest and highest values for each barrier. According to the mean results, which range from 2.56 to 2.62, the majority of respondents either deny or are unconcerned about the significant barriers to exercise. The SD numbers, which range from 1.37 to 1.41 and demonstrate a substantial variance in replies, suggest that some persons face more difficulties than others. The positive skewness of all categories (range 0.92 to 1.01) showed that most respondents reported mild levels of perceived problems, while a smaller minority reported significant issues. The statement with the highest skewness (1.01) was “I struggle to start or maintain exercise routines, even after consuming sports media content,” indicating that while most respondents think they can exercise, some still find it difficult to stay motivated. Responses appear to be equally distributed over the scale, as indicated by the kurtosis values (which range from 0.05 to 0.32), which suggest distributions around normal. “I struggle to start or maintain exercise routines, even after consuming sports media content,” had the highest kurtosis (0.32), indicating that responses are fairly clustered around a neutral position.

**Table 4:** Descriptive statistics for perceived barriers to exercise.

Perceived barriers to exercise	Mean	SD	Min	Max	Skew	Kurtosis
My lack of time prevents me from exercising regularly, despite the influence of media.	2.58	1.41	1	6	0.97	0.16
Financial constraints limit my ability to participate in fitness activities portrayed in sports media.	2.56	1.38	1	6	1	0.25
I struggle to start or maintain exercise routines, even after consuming sports media content.	2.56	1.37	1	6	1.01	0.32
The fitness routines shown in Chinese sports media seem too difficult for me to achieve.	2.6	1.4	1	6	0.96	0.12
External factors (e.g., work or family responsibilities) make it challenging to act on media messages about exercise.	2.62	1.4	1	6	0.93	0.05
Competing priorities often make it hard for me to focus on exercise, despite its media-promoted importance.	2.61	1.38	1	6	0.92	0.09

Table 5 displays the descriptive data for Media Influence on Exercise Habits, which assesses how much exposure to Chinese sports media influences people's exercise routines. The table includes the mean, skewness, kurtosis, lowest and highest values, and SD for each statement measuring media influence. A majority of respondents reported media exposure as being associated with greater exercise motivation, as indicated by the mean values, which range from 4.37 to 4.47. According to the SD numbers, which vary from 1.33 to 1.4, the media less effect some people than others, even if many people feel that way. The negative skewness of all the items (ranging from −0.92 to −1.01) indicates that the majority of respondents agreed that the media had a significant impact on exercise. “I actively seek sports media content for exercise inspiration and motivation,” which has the largest skewness (−1.01), suggests that a significant percentage of respondents frequently use media to find fitness-related information. Kurtosis scores (which range from 0.11 to 0.36) indicate that answers are almost normal, indicating that people's degrees of agreement are dispersed equally. The statement, “I actively seek sports media content for exercise inspiration and motivation,” had the highest kurtosis (0.36), indicating a somewhat more concentrated dispersion of answers around the mean.

**Table 5:** Descriptive statistics for Media influence on exercise habits.

Media influence on exercise habits	Mean	SD	Min	Max	Skew	Kurtosis
My exercise habits have improved due to the influence of Chinese sports media.	4.37	1.39	1	6	−0.95	0.16
I actively seek sports media content for exercise inspiration and motivation.	4.47	1.37	1	6	−1.01	0.36
I am likely to follow exercise routines promoted in Chinese sports media.	4.43	1.33	1	6	−0.92	0.3
The frequency of consuming Chinese sports media influences my commitment to regular exercise.	4.45	1.37	1	6	−0.97	0.22
Media messages about exercise help me prioritize physical activity in my daily routine.	4.39	1.38	1	6	−0.92	0.11
Consuming Chinese sports media strengthens my fitness goals and ambitions.	4.41	1.4	1	6	−0.98	0.23

Table 6 displays the results of the evaluation of the Structural Equation Model (SEM) model. The relationships between self-efficacy, perceived barriers to exercise, perceived benefits of exercise, perceived vulnerability to health risks, and the influence of media on exercise habits were examined using the SEM. The Chi-square test, the SRMR, the RMSEA and its CI, and the CFI are some of the noteworthy fit indices in the table. These measures make it easier to assess how well the suggested model explains the data that has been seen. The chi-square test result with 540 degrees of freedom is 543.371, and the *p*-value that goes with it is 0.451. Since there is no significant difference between the model and the data, the *p*-value is greater than 0.05, which suggests that the proposed model appropriately reflects the relationships between the variables. However, because the Chi-square test is sensitive to sample size, other fit indices provide additional insights into the model's adequacy.

**Table 6:** Model evaluation for structural equation model fit.

Fit index	Value
Chi-square test (DF = 540)	543.371
*p*-value	0.451
CFI	1.000
RMSEA	0.002
90% CI for RMSEA	[0.000, 0.010]
SRMR	0.022

The CFI is 1.000, which is well above the commonly accepted threshold of 0.95. This suggests an excellent fit, indicating the proposed model explains observed associations between latent constructs with minimal error. However, due to the cross-sectional nature, these relationships should be interpreted as correlational. The 90% CI for the RMSEA is [0.000, 0.010], and it is 0.002. A good fit is defined as an RMSEA value less than 0.05, while an acceptable fit is defined as a value less than 0.08. The computed RMSEA value, which shows that there is very low approximation error in the model, further supports the model's superb fit to the data. The SRMR is much below the recommended limit of 0.08 at 0.022. A lower SRMR score, which shows that the residuals—differences between observed and expected correlations—are minimal, suggests that the model fits the data well.

## 4 Discussion

This study examined how perceived health beliefs, framed by Chinese digital sports media, correlate with young adults' self-reported motivation to exercise. Using the HBM as a guiding framework, we tested the association between media exposure and four key constructs: susceptibility, benefits, barriers, and self-efficacy. While the findings largely support HBM assumptions, the study also illustrates how media ecosystems in China influence belief formation in unique ways. These insights should be viewed as preliminary and descriptive, given the study's cross-sectional design. Perceived susceptibility is positively associated with self-reported exercise motivation, with young individuals who perceive more significant health risks from inactive lives engaging in more exercise. The SEM findings (Table 6) indicate that individuals who report higher awareness of health risks also report greater motivation to exercise (8). These findings support Darlow et al. (26) discovery that those with a higher perceived risk of obesity and cardiovascular disease were efficient. Ojangba et al. (27) discovered that young people's awareness of chronic illnesses such as hypertension and diabetes predicted exercise participation.

China values state-led health efforts. The “Healthy China 2030” campaign has promoted awareness of lifestyle diseases through sports media, social media influencers, and television public service announcements (28). Health information is frequently disseminated, which could explain the significant link between perceived vulnerability and exercise participation in this study. According to Xiang et al. (29), Chinese sports media uniquely impacts common attitudes about exercise, frequently promoting government-backed health narratives and disease prevention through physical activity. Unlike Western applications of the HBM, which often emphasize individual autonomy in health decisions, our findings suggest that Chinese sports media embeds health behaviors within a broader context of civic responsibility and national fitness identity. This state-structured narrative may alter how perceived susceptibility and benefits are internalized. While the data broadly support the HBM, many studies show that awareness alone does not always lead to action (8, 30). Barnes, Newman, and Keenan (31) discovered that those who were aware of their health issues but lacked motivation or self-efficacy were less likely to exercise. To fully understand media-driven behavior changes, self-efficacy must be considered.

While exercise is promoted for mental and physical health benefits, a significant portion of respondents (49.1%) also cited appearance as a motivator (32, 33). This aligns with prior research showing Chinese sports media often emphasizes ideal body types. While such portrayals may increase perceived benefits, they also risk promoting narrow beauty standards (34). Future studies should examine how exposure to appearance-focused content affects self-efficacy and long-term adherence, especially when comparisons with idealized influencers generate discouragement rather than motivation.

Chinese sports media stresses aesthetic benefits, which may lead to body dissatisfaction and workout anxiety. According to Jackson et al. (35), exposure to idealized fitness images can lead to exercise avoidance or poor weight management. The HBM contends that perceived benefits lead to positive behavioral change; nevertheless, this study demonstrates that media can motivate exercise and cause body dissatisfaction. Regardless of these issues, sports media can be commended for promoting exercise's mental health benefits (8). This study validates the findings of Qu et al. (36), who discovered that media-driven stress reduction and mental health messaging increased young adult exercise motivation. China is embracing mindfulness-based fitness programs, such as yoga and tai chi, as aesthetic considerations give way to a more holistic view of exercise benefits.

Self-efficacy influenced exercise habits, supporting Linge et al. (37) assertion that confidence in one's ability to exercise is a significant predictor of health behavior. This study validates Tricás-Vidal et al. (38) findings that young adults who watch sports media influencers and fitness videos had higher self-efficacy and exercise adherence levels. This study found that media consumption is positively associated with higher self-efficacy, although the cross-sectional design limits interpretation of directionality. Zhang et al. (39) discovered that following online fitness networks and influencers boosts self-efficacy by offering instructional guidance, success stories, and peer support.

Fitness influencers on Douyin (TikTok) and Weibo assist young Chinese adults in overcoming training obstacles. Aguilar and Arbaiza (40) discovered that Chinese sports influencers who disclose their fitness adventures, challenges, and accomplishments increase followers' confidence in their fitness ambitions. This study supports these findings, revealing how digital sports media influences self-efficacy. It is important to note that the study cannot determine whether media consumption causes increased self-efficacy. The direction of influence also reflects self-selection, where more confident individuals are more likely to engage with fitness content. The HBM encourages self-efficacy in behavior modification, but it ignores the effects of media intake on social comparisons. Fardouly et al. (41) observed that negative social comparisons with fitness influencers can reduce self-efficacy and discourage participation. This study found no negative consequences, but future research should investigate whether unrealistic fitness images in Chinese media can affect self-efficacy over time.

Perceived barriers, such as time, cost, and competing priorities, were negatively associated with exercise behavior. These findings are consistent with HBM literature, suggesting that as perceived obstacles increase, motivation to act decreases (42). These findings reflect Zhang et al. (43) conclusion that Chinese young adults cite scholastic and employment obligations as substantial impediments to regular exercise. However, Western research suggests that a lack of fitness facilities is a significant hindrance. The urban-centric structure of Chinese exercise culture makes gym memberships and organized fitness programs prohibitively expensive, limiting participation (44).

This study found that media exposure increases self-efficacy and motivation while lowering perceived barriers. Like Xiang et al. (29), Chinese sports media content that addresses common exercise challenges such as home workouts, time-saving routines, and cost-effective fitness solutions encourages participation. Although media can lessen psychological barriers, it cannot eliminate structural ones. Aside from media influence, Gao et al. (45) proposed legislative changes, infrastructure upgrades, and public fitness space accessibility to address exercise hurdles. This study demonstrates how Chinese sports media dramatically influences young adults' exercise habits via HBM mechanisms. The findings support international literature while emphasizing Chinese media culture. Media-driven perceived susceptibility, benefits, and self-efficacy increase exercise motivation, but perceived barriers discourage it (4, 45). Moreover, while digital media may reduce psychological barriers through motivational content and low-cost workout ideas, it cannot fully compensate for structural constraints such as limited access to facilities in rural or low-income areas.

## 5 Conclusion

This study examined how Chinese sports media relate to exercise motivation among young adults through the framework of the HBM. The results demonstrate that exposure to sports media is positively associated with three core HBM constructs: perceived susceptibility to health risks, perceived benefits of exercise, and self-efficacy. Conversely, perceived barriers to exercise show a negative association with motivation to engage in physical activity. Among the four factors, self-efficacy had the strongest positive correlation with exercise behavior. These associations were confirmed through Structural Equation Modeling, which revealed excellent model fit, supporting the internal consistency and reliability of the theoretical relationships proposed. Importantly, this research is based on a cross-sectional survey design and cannot establish causal links between media exposure and exercise behavior. The findings reflect statistically significant correlations rather than directional effects. Therefore, any interpretation of media influence should be confined to observed patterns of association.

Additionally, the study identifies that while media messaging may enhance motivation and self-efficacy, structural barriers, such as financial costs, limited time, or access to facilities, remain critical obstacles. These findings suggest that media alone cannot drive behavior change unless complemented by broader systemic support. The conclusion here focuses solely on summarizing the empirical outcomes of the research without conflating them with the study's theoretical or practical value. Future work should build on these findings using longitudinal and experimental designs to explore how sustained media exposure might influence behavior over time.

### 5.1 Implications and contributions

This study contributes to health communication, behavioral psychology, and media studies by applying the Health Belief Model within China's digital sports media ecosystem. Rather than extending the theory, this research contextualizes it in a media landscape shaped by government narratives and social media influencers. By situating HBM constructs in this environment, the study offers culturally specific insights into how media may shape exercise-related beliefs and attitudes. Practically, the findings offer guidance for public health practitioners, media campaign designers, and digital influencers. Content that enhances perceived benefits and self-efficacy, while addressing common barriers, can potentially strengthen youth engagement with physical activity. Additionally, the study underscores the need to balance aspirational media messaging with inclusive, attainable representations of fitness to avoid demotivating comparisons.

### 5.2 Future suggestions

Media influence should be investigated longitudinally to determine whether initial exposure maintains exercise engagement or decreases motivation. Long-term behavioral change mechanisms can be better understood by examining how media influences health attitudes. Different media setups need cross-cultural comparisons between China and other regions. Comparing Western sports media platforms (Instagram, YouTube, and ESPN) and Chinese platforms (Weibo, Douyin, CCTV-5) in terms of influence on exercise habits would provide worldwide insights. Media exposure could also be altered in experimental studies to examine its impact on exercise. Controlled interventions comparing health-risk framing, self-efficacy reinforcement, and social influence strategies could help shape public health programs. Finally, wearable fitness technologies and big data analytics may increase the objectivity and precision of exercise engagement measurement in future studies. AI-powered analytics and real-time behavioral tracking may improve media-based physical activity assessments. Future research should prioritize longitudinal tracking and experimental design to test how media exposure sustains, or fails to sustain, behavioral change over time.

## Data Availability

The raw data supporting the conclusions of this article will be made available by the authors, without undue reservation.
