# Supplementary material for: Investigating the role of Chinese sports media in shaping young adults’ exercise habits through the lens of the health belief model
Source: Front Sports Act Living. 2025 Sep 22;7:1600486. doi: 10.3389/fspor.2025.1600486 (PMC12497700; doi:10.3389/fspor.2025.1600486)
Supplement: Supplementary file 1 [file Datasheet1.docx]

**Appendix**

| **What is your age?** | | | | | |
| --- | --- | --- | --- | --- | --- |
|  | | Frequency | Percent | Valid Percent | Cumulative Percent |
| Valid | Below 18 | 58 | 4.8 | 4.8 | 4.8 |
|  | 18-24 | 661 | 54.1 | 54.1 | 58.9 |
|  | 25-30 | 371 | 30.4 | 30.4 | 89.3 |
|  | Above 30 | 131 | 10.7 | 10.7 | 100.0 |
|  | Total | 1221 | 100.0 | 100.0 |  |

| **What is your gender?** | | | | | |
| --- | --- | --- | --- | --- | --- |
|  | | Frequency | Percent | Valid Percent | Cumulative Percent |
| Valid | Male | 652 | 53.4 | 53.4 | 53.4 |
|  | Female | 569 | 46.6 | 46.6 | 100.0 |
|  | Total | 1221 | 100.0 | 100.0 |  |

| **What is the highest level of education you have completed?** | | | | | |
| --- | --- | --- | --- | --- | --- |
|  | | Frequency | Percent | Valid Percent | Cumulative Percent |
| Valid | High School | 58 | 4.8 | 4.8 | 4.8 |
|  | Bachelor's Degree | 814 | 66.7 | 66.7 | 71.4 |
|  | Master's Degree | 287 | 23.5 | 23.5 | 94.9 |
|  | P.hD or Higher | 62 | 5.1 | 5.1 | 100.0 |
|  | Total | 1221 | 100.0 | 100.0 |  |

| **How often do you engage in physical exercise?** | | | | | |
| --- | --- | --- | --- | --- | --- |
|  | | Frequency | Percent | Valid Percent | Cumulative Percent |
| Valid | Rarely (less than once a week) | 242 | 19.8 | 19.8 | 19.8 |
|  | Occasionally (1–2 times a week) | 373 | 30.5 | 30.5 | 50.4 |
|  | Often (3–5 times a week) | 498 | 40.8 | 40.8 | 91.2 |
|  | Very Often (daily) | 108 | 8.8 | 8.8 | 100.0 |
|  | Total | 1221 | 100.0 | 100.0 |  |

| **How often do you consume Chinese sports media (e.g., TV, online platforms, or social media)?** | | | | | |
| --- | --- | --- | --- | --- | --- |
|  | | Frequency | Percent | Valid Percent | Cumulative Percent |
| Valid | Rarely | 119 | 9.7 | 9.7 | 9.7 |
|  | Occasionally | 613 | 50.2 | 50.2 | 60.0 |
|  | Often | 366 | 30.0 | 30.0 | 89.9 |
|  | Very Often | 123 | 10.1 | 10.1 | 100.0 |
|  | Total | 1221 | 100.0 | 100.0 |  |

| **Do you have consistent access to Chinese sports media platforms?** | | | | | |
| --- | --- | --- | --- | --- | --- |
|  | | Frequency | Percent | Valid Percent | Cumulative Percent |
| Valid | Yes‎ | 921 | 75.4 | 75.4 | 75.4 |
|  | No | 300 | 24.6 | 24.6 | 100.0 |
|  | Total | 1221 | 100.0 | 100.0 |  |

| **What is your primary motivation for engaging in physical activities?** | | | | | |
| --- | --- | --- | --- | --- | --- |
|  | | Frequency | Percent | Valid Percent | Cumulative Percent |
| Valid | Health reasons | 430 | 35.2 | 35.2 | 35.2 |
|  | Improving appearance | 599 | 49.1 | 49.1 | 84.3 |
|  | Social interaction | 68 | 5.6 | 5.6 | 89.8 |
|  | Following trends | 124 | 10.2 | 10.2 | 100.0 |
|  | Total | 1221 | 100.0 | 100.0 |  |

**Descriptive Statistics of the items**

vars n mean sd median trimmed mad min max range skew kurtosis se

PS1 1 1221 4.81 1.44 5 5.05 1.48 1 6 5 -1.06 0.16 0.04

PS2 2 1221 4.40 1.40 5 4.58 1.48 1 6 5 -0.96 0.21 0.04

PS3 3 1221 4.72 1.51 5 4.97 1.48 1 6 5 -1.02 0.00 0.04

PS4 4 1221 4.42 1.35 5 4.58 1.48 1 6 5 -0.95 0.27 0.04

PS5 5 1221 4.43 1.36 5 4.61 1.48 1 6 5 -1.00 0.37 0.04

PS6 6 1221 4.46 1.31 5 4.63 1.48 1 6 5 -0.98 0.43 0.04

PB1 7 1221 4.77 1.48 5 5.02 1.48 1 6 5 -1.06 0.14 0.04

PB2 8 1221 4.44 1.36 5 4.62 1.48 1 6 5 -0.96 0.27 0.04

PB3 9 1221 4.39 1.39 5 4.56 1.48 1 6 5 -0.94 0.16 0.04

PB4 10 1221 4.39 1.37 5 4.57 1.48 1 6 5 -0.97 0.29 0.04

PB5 11 1221 4.36 1.34 5 4.52 1.48 1 6 5 -0.91 0.17 0.04

PB6 12 1221 4.41 1.33 5 4.58 1.48 1 6 5 -0.97 0.32 0.04

SE1 13 1221 4.39 1.40 5 4.56 1.48 1 6 5 -0.92 0.10 0.04

SE2 14 1221 4.45 1.33 5 4.62 1.48 1 6 5 -1.03 0.44 0.04

SE3 15 1221 4.44 1.35 5 4.61 1.48 1 6 5 -0.98 0.34 0.04

SE4 16 1221 4.46 1.33 5 4.63 1.48 1 6 5 -0.94 0.32 0.04

SE5 17 1221 4.46 1.34 5 4.63 1.48 1 6 5 -0.98 0.31 0.04

SE6 18 1221 4.39 1.34 5 4.54 1.48 1 6 5 -0.89 0.21 0.04

BE1 19 1221 2.58 1.41 2 2.40 1.48 1 6 5 0.97 0.16 0.04

BE2 20 1221 2.56 1.38 2 2.39 1.48 1 6 5 1.00 0.25 0.04

BE3 21 1221 2.56 1.37 2 2.38 1.48 1 6 5 1.01 0.32 0.04

BE4 22 1221 2.60 1.40 2 2.43 1.48 1 6 5 0.96 0.12 0.04

BE5 23 1221 2.62 1.40 2 2.46 1.48 1 6 5 0.93 0.05 0.04

BE6 24 1221 2.61 1.38 2 2.45 1.48 1 6 5 0.92 0.09 0.04

MI1 25 1221 4.37 1.39 5 4.53 1.48 1 6 5 -0.95 0.16 0.04

MI2 26 1221 4.47 1.37 5 4.66 1.48 1 6 5 -1.01 0.36 0.04

MI3 27 1221 4.43 1.33 5 4.59 1.48 1 6 5 -0.92 0.30 0.04

MI4 28 1221 4.45 1.37 5 4.62 1.48 1 6 5 -0.97 0.22 0.04

MI5 29 1221 4.39 1.38 5 4.55 1.48 1 6 5 -0.92 0.11 0.04

MI6 30 1221 4.41 1.40 5 4.59 1.48 1 6 5 -0.98 0.23 0.04

**Reliability analysis**

Call: psych::alpha(x = data %>% select(PS1, PS2, PS3, PS4, PS5, PS6,

PB1, PB2, PB3, PB4, PB5, PB6, SE1, SE2, SE3, SE4, SE5, SE6,

BE1, BE2, BE3, BE4, BE5, BE6, MI1, MI2, MI3, MI4, MI5, MI6),

check.keys = TRUE)

raw_alpha std.alpha G6(smc) average_r S/N ase mean sd median_r

0.94 0.94 0.94 0.35 16 0.0024 4.5 0.84 0.35

95% confidence boundaries

lower alpha upper

Feldt 0.94 0.94 0.95

Duhachek 0.94 0.94 0.95

Reliability if an item is dropped:

raw_alpha std.alpha G6(smc) average_r S/N alpha se var.r med.r

PS1 0.94 0.94 0.94 0.35 15 0.0025 0.00057 0.35

PS2 0.94 0.94 0.94 0.35 16 0.0025 0.00070 0.35

PS3 0.94 0.94 0.94 0.35 16 0.0025 0.00069 0.35

PS4 0.94 0.94 0.94 0.35 16 0.0025 0.00071 0.35

PS5 0.94 0.94 0.94 0.35 16 0.0025 0.00068 0.35

PS6 0.94 0.94 0.94 0.35 16 0.0025 0.00069 0.35

PB1 0.94 0.94 0.94 0.35 16 0.0025 0.00067 0.35

PB2 0.94 0.94 0.94 0.35 16 0.0025 0.00069 0.35

PB3 0.94 0.94 0.94 0.35 16 0.0025 0.00068 0.35

PB4 0.94 0.94 0.94 0.35 16 0.0025 0.00069 0.35

PB5 0.94 0.94 0.94 0.35 16 0.0025 0.00066 0.35

PB6 0.94 0.94 0.94 0.35 16 0.0025 0.00070 0.35

SE1 0.94 0.94 0.94 0.35 16 0.0025 0.00071 0.35

SE2 0.94 0.94 0.94 0.35 16 0.0025 0.00065 0.35

SE3 0.94 0.94 0.94 0.35 16 0.0025 0.00066 0.35

SE4 0.94 0.94 0.94 0.35 16 0.0025 0.00070 0.35

SE5 0.94 0.94 0.94 0.35 16 0.0025 0.00068 0.35

SE6 0.94 0.94 0.94 0.35 16 0.0025 0.00070 0.35

BE1- 0.94 0.94 0.94 0.35 16 0.0025 0.00071 0.35

BE2- 0.94 0.94 0.94 0.35 16 0.0025 0.00070 0.35

BE3- 0.94 0.94 0.94 0.35 16 0.0025 0.00069 0.35

BE4- 0.94 0.94 0.94 0.35 16 0.0025 0.00070 0.35

BE5- 0.94 0.94 0.94 0.35 16 0.0025 0.00069 0.35

BE6- 0.94 0.94 0.94 0.35 16 0.0025 0.00070 0.35

MI1 0.94 0.94 0.94 0.35 16 0.0025 0.00068 0.35

MI2 0.94 0.94 0.94 0.35 16 0.0025 0.00069 0.35

MI3 0.94 0.94 0.94 0.35 16 0.0025 0.00070 0.35

MI4 0.94 0.94 0.94 0.35 16 0.0025 0.00070 0.35

MI5 0.94 0.94 0.94 0.35 16 0.0025 0.00069 0.35

MI6 0.94 0.94 0.94 0.35 16 0.0025 0.00070 0.35

Item statistics

n raw.r std.r r.cor r.drop mean sd

PS1 1221 0.68 0.68 0.67 0.65 4.8 1.4

PS2 1221 0.59 0.59 0.57 0.55 4.4 1.4

PS3 1221 0.63 0.63 0.61 0.59 4.7 1.5

PS4 1221 0.62 0.62 0.60 0.58 4.4 1.4

PS5 1221 0.64 0.64 0.63 0.61 4.4 1.4

PS6 1221 0.62 0.62 0.60 0.58 4.5 1.3

PB1 1221 0.64 0.64 0.62 0.61 4.8 1.5

PB2 1221 0.61 0.61 0.59 0.58 4.4 1.4

PB3 1221 0.59 0.59 0.57 0.55 4.4 1.4

PB4 1221 0.61 0.61 0.59 0.57 4.4 1.4

PB5 1221 0.58 0.59 0.57 0.55 4.4 1.3

PB6 1221 0.59 0.59 0.57 0.55 4.4 1.3

SE1 1221 0.61 0.61 0.60 0.58 4.4 1.4

SE2 1221 0.64 0.64 0.63 0.61 4.5 1.3

SE3 1221 0.58 0.58 0.56 0.55 4.4 1.3

SE4 1221 0.61 0.61 0.59 0.57 4.5 1.3

SE5 1221 0.60 0.60 0.58 0.57 4.5 1.3

SE6 1221 0.60 0.60 0.58 0.57 4.4 1.3

BE1- 1221 0.62 0.62 0.60 0.58 4.4 1.4

BE2- 1221 0.60 0.60 0.58 0.56 4.4 1.4

BE3- 1221 0.62 0.62 0.60 0.58 4.4 1.4

BE4- 1221 0.60 0.60 0.58 0.56 4.4 1.4

BE5- 1221 0.63 0.63 0.61 0.59 4.4 1.4

BE6- 1221 0.59 0.59 0.57 0.55 4.4 1.4

MI1 1221 0.58 0.58 0.56 0.54 4.4 1.4

MI2 1221 0.62 0.62 0.61 0.59 4.5 1.4

MI3 1221 0.60 0.60 0.58 0.56 4.4 1.3

MI4 1221 0.60 0.60 0.58 0.57 4.4 1.4

MI5 1221 0.60 0.60 0.58 0.56 4.4 1.4

MI6 1221 0.60 0.60 0.58 0.56 4.4 1.4

Non missing response frequency for each item

1 2 3 4 5 6 miss

PS1 0.04 0.06 0.08 0.18 0.17 0.47 0

PS2 0.06 0.06 0.08 0.21 0.37 0.21 0

PS3 0.05 0.05 0.09 0.17 0.18 0.45 0

PS4 0.05 0.07 0.08 0.23 0.37 0.20 0

PS5 0.05 0.06 0.08 0.22 0.39 0.21 0

PS6 0.05 0.06 0.09 0.21 0.40 0.20 0

PB1 0.05 0.06 0.07 0.19 0.17 0.47 0

PB2 0.05 0.06 0.09 0.20 0.38 0.22 0

PB3 0.06 0.07 0.08 0.22 0.37 0.21 0

PB4 0.06 0.06 0.09 0.22 0.38 0.20 0

PB5 0.05 0.07 0.10 0.21 0.39 0.18 0

PB6 0.05 0.07 0.08 0.21 0.40 0.19 0

SE1 0.06 0.07 0.08 0.21 0.37 0.21 0

SE2 0.05 0.07 0.08 0.20 0.41 0.19 0

SE3 0.05 0.06 0.09 0.21 0.38 0.21 0

SE4 0.04 0.06 0.09 0.22 0.37 0.22 0

SE5 0.05 0.06 0.09 0.20 0.39 0.21 0

SE6 0.05 0.06 0.09 0.24 0.36 0.20 0

BE1 0.22 0.36 0.22 0.05 0.08 0.06 0

BE2 0.21 0.39 0.21 0.06 0.08 0.05 0

BE3 0.21 0.39 0.22 0.05 0.08 0.05 0

BE4 0.21 0.38 0.21 0.06 0.08 0.06 0

BE5 0.20 0.38 0.21 0.06 0.09 0.05 0

BE6 0.20 0.37 0.22 0.06 0.09 0.05 0

MI1 0.06 0.07 0.07 0.22 0.38 0.19 0

MI2 0.05 0.06 0.08 0.19 0.39 0.22 0

MI3 0.05 0.07 0.08 0.24 0.36 0.21 0

MI4 0.05 0.07 0.08 0.20 0.39 0.22 0

MI5 0.05 0.08 0.07 0.22 0.37 0.21 0

MI6 0.06 0.07 0.07 0.22 0.38 0.21 0

**Summary of SEM result**

lavaan 0.6-19 ended normally after 87 iterations

Estimator ML

Optimization method NLMINB

Number of model parameters 75

Number of observations 1221

Model Test User Model:

Test statistic 543.371

Degrees of freedom 540

P-value (Chi-square) 0.451

Model Test Baseline Model:

Test statistic 12940.175

Degrees of freedom 585

P-value 0.000

User Model versus Baseline Model:

Comparative Fit Index (CFI) 1.000

Tucker-Lewis Index (TLI) 1.000

Loglikelihood and Information Criteria:

Loglikelihood user model (H0) -57470.316

Loglikelihood unrestricted model (H1) -57198.631

Akaike (AIC) 115090.632

Bayesian (BIC) 115473.689

Sample-size adjusted Bayesian (SABIC) 115235.458

Root Mean Square Error of Approximation:

RMSEA 0.002

90 Percent confidence interval - lower 0.000

90 Percent confidence interval - upper 0.010

P-value H_0: RMSEA <= 0.050 1.000

P-value H_0: RMSEA >= 0.080 0.000

Standardized Root Mean Square Residual:

SRMR 0.022

Parameter Estimates:

Standard errors Standard

Information Expected

Information saturated (h1) model Structured

Latent Variables:

Estimate Std.Err z-value P(>|z|) Std.lv Std.all

Perceived_Susceptibility =~

PS1 1.000 0.958 0.664

PS2 0.825 0.045 18.421 0.000 0.790 0.565

PS3 0.957 0.049 19.670 0.000 0.917 0.608

PS4 0.844 0.044 19.378 0.000 0.808 0.598

PS5 0.888 0.044 20.240 0.000 0.850 0.627

PS6 0.822 0.042 19.505 0.000 0.788 0.602

Perceived_Benefits =~

PB1 1.000 0.909 0.616

PB2 0.879 0.048 18.298 0.000 0.799 0.588

PB3 0.858 0.049 17.567 0.000 0.780 0.560

PB4 0.873 0.048 18.048 0.000 0.793 0.578

PB5 0.828 0.047 17.576 0.000 0.753 0.560

PB6 0.821 0.047 17.619 0.000 0.747 0.562

Self_Efficacy =~

SE1 1.000 0.834 0.594

SE2 0.999 0.054 18.496 0.000 0.833 0.629

SE3 0.910 0.054 16.989 0.000 0.759 0.563

SE4 0.937 0.053 17.610 0.000 0.781 0.590

SE5 0.935 0.054 17.408 0.000 0.780 0.581

SE6 0.939 0.054 17.496 0.000 0.783 0.585

Barriers_to_Exercise =~

BE1 1.000 0.849 0.601

BE2 0.946 0.054 17.464 0.000 0.803 0.581

BE3 0.971 0.054 17.956 0.000 0.824 0.602

BE4 0.954 0.055 17.391 0.000 0.809 0.578

BE5 1.007 0.055 18.175 0.000 0.854 0.611

BE6 0.932 0.054 17.244 0.000 0.791 0.572

Exercise_Habits =~

MI1 1.000 0.775 0.558

MI2 1.064 0.062 17.150 0.000 0.824 0.604

MI3 0.994 0.060 16.696 0.000 0.771 0.581

MI4 1.023 0.061 16.658 0.000 0.793 0.579

MI5 1.023 0.062 16.558 0.000 0.793 0.574

MI6 1.041 0.063 16.618 0.000 0.807 0.577

Regressions:

Estimate Std.Err z-value P(>|z|) Std.lv Std.all

Exercise_Habits ~

Prcvd_Sscptblt 0.199 1.075 0.185 0.853 0.246 0.246

Perceivd_Bnfts 0.075 0.471 0.159 0.874 0.088 0.088

Self_Efficacy 0.709 0.745 0.952 0.341 0.763 0.763

Barrrs_t_Exrcs 0.082 0.738 0.111 0.912 0.089 0.089

Age 0.006 0.020 0.282 0.778 0.007 0.005

Gender -0.006 0.028 -0.230 0.818 -0.008 -0.004

Education_Levl -0.005 0.023 -0.207 0.836 -0.006 -0.004

Exercis_Frqncy 0.010 0.015 0.685 0.493 0.013 0.012

Media_Consmptn 0.016 0.017 0.934 0.350 0.021 0.017

Covariances:

Estimate Std.Err z-value P(>|z|) Std.lv Std.all

Perceived_Susceptibility ~~

Perceivd_Bnfts 0.883 0.057 15.545 0.000 1.015 1.015

Self_Efficacy 0.804 0.053 15.152 0.000 1.007 1.007

Barrrs_t_Exrcs -0.814 0.054 -15.196 0.000 -1.002 -1.002

Perceived_Benefits ~~

Self_Efficacy 0.775 0.053 14.720 0.000 1.022 1.022

Barrrs_t_Exrcs -0.786 0.053 -14.772 0.000 -1.019 -1.019

Self_Efficacy ~~

Barrrs_t_Exrcs -0.698 0.049 -14.329 0.000 -0.986 -0.986

Variances:

Estimate Std.Err z-value P(>|z|) Std.lv Std.all

.PS1 1.165 0.050 23.211 0.000 1.165 0.559

.PS2 1.329 0.056 23.919 0.000 1.329 0.680

.PS3 1.436 0.061 23.678 0.000 1.436 0.631

.PS4 1.176 0.050 23.741 0.000 1.176 0.643

.PS5 1.116 0.047 23.539 0.000 1.116 0.607

.PS6 1.092 0.046 23.714 0.000 1.092 0.638

.PB1 1.352 0.057 23.741 0.000 1.352 0.621

.PB2 1.207 0.050 23.925 0.000 1.207 0.654

.PB3 1.334 0.055 24.073 0.000 1.334 0.687

.PB4 1.252 0.052 23.980 0.000 1.252 0.665

.PB5 1.240 0.052 24.072 0.000 1.240 0.686

.PB6 1.209 0.050 24.064 0.000 1.209 0.684

.SE1 1.274 0.054 23.659 0.000 1.274 0.647

.SE2 1.062 0.045 23.388 0.000 1.062 0.605

.SE3 1.242 0.052 23.850 0.000 1.242 0.683

.SE4 1.145 0.048 23.691 0.000 1.145 0.652

.SE5 1.193 0.050 23.747 0.000 1.193 0.663

.SE6 1.181 0.050 23.723 0.000 1.181 0.658

.BE1 1.277 0.054 23.519 0.000 1.277 0.639

.BE2 1.267 0.054 23.658 0.000 1.267 0.663

.BE3 1.197 0.051 23.509 0.000 1.197 0.638

.BE4 1.307 0.055 23.678 0.000 1.307 0.666

.BE5 1.223 0.052 23.434 0.000 1.223 0.626

.BE6 1.289 0.054 23.717 0.000 1.289 0.673

.MI1 1.327 0.055 23.944 0.000 1.327 0.688

.MI2 1.186 0.050 23.664 0.000 1.186 0.636

.MI3 1.167 0.049 23.817 0.000 1.167 0.663

.MI4 1.247 0.052 23.828 0.000 1.247 0.665

.MI5 1.280 0.054 23.857 0.000 1.280 0.670

.MI6 1.304 0.055 23.839 0.000 1.304 0.667

Prcvd_Sscptblt 0.917 0.072 12.751 0.000 1.000 1.000

Perceivd_Bnfts 0.826 0.071 11.654 0.000 1.000 1.000

Self_Efficacy 0.696 0.063 11.088 0.000 1.000 1.000

Barrrs_t_Exrcs 0.720 0.064 11.194 0.000 1.000 1.000

.Exercise_Habts -0.013 0.013 -1.014 0.310 -0.022 -0.022
